# Intraindividual variability in non-household contacts: a German longitudinal study, April 2020–December 2021

**DOI:** 10.1186/s12879-026-12940-4

**Published:** 2026-02-21

**Authors:** Chao Xu, Aleksandr Bryzgalov, Johannes Horn, Andrzej K. Jarynowski, Vitaly Belik, Veronika K Jaeger, André Karch, Huynh Thi Phuong, Janik Suer, Marlli Zambrano, Steven Schulz, Alejandra Rincón Hidalgo, Ashish Thampi, Richard Pastor, Rafael Mikolajczyk

**Affiliations:** 1 Institute for Medical Epidemiology, Biometrics, and Informatics, Martin Luther University Halle-Wittenberg, Halle, Germany; 2 Institute of Epidemiology and Social Medicine, University of Münster, Münster, Germany; 3 System Modelling Group, Institute of Veterinary Epidemiology and Biostatistics, Freie Universität Berlin, Berlin, Germany; 4 Machine Learning Unit, Department of Engineering, NET CHECK GmbH, Berlin, Germany; 5 Interdisciplinary Research Institute, Wroclaw, Poland; 6 Division of Healthcare Innovation, Faculty of Health Sciences, Wrocław Medical University, Wroclaw, Poland

**Keywords:** Contact rate, Contact variability, Infectious disease modelling, Vaccination, Policy stringency

## Abstract

**Background:**

Day-to-day variability in social contacts can shape transmission dynamics yet is rarely quantified. We aimed to quantify intraindividual variability (IIV) in non-household contacts during the COVID-19 pandemic in Germany and to assess its associations with sociodemographic characteristics, vaccination, and policy stringency.

**Methods:**

We analyzed longitudinal contact survey data with 33 waves between April 2020 and December 2021, including 7,845 participants and 59,462 observations. Pearson residuals from a mixed-effects negative binomial model were used to derive the within-person standard deviation (riSD) for participants with at least two observations, as a proxy of IIV. Gamma regression models with log link were fitted to estimate mean ratios (MR).

**Results:**

Children and adolescents aged 0–17 years showed higher riSD than other age groups (MR = 1.13, 95% CI 1.09–1.16). Participants living in households with three or more members had higher riSD than those living alone (1.05, 95% CI 1.02–1.07). Retired individuals, homemakers, the unemployed, and students exhibited lower riSD than employed participants. Regarding COVID-19 vaccination, compared with the pre-vaccination window (− 100 to 0 days), riSD was higher in the post-vaccination window (1 to 100 days after the first COVID-19 vaccination dose) (1.13, 95% CI 1.06–1.20). Weaker policy stringency was strongly associated with higher riSD (1.36, 95% CI 1.32–1.39).

**Conclusions:**

IIV in non-household contacts was shaped by age, household composition, employment status, vaccination status, and policy context. Analyses relying solely on average contact numbers may misrepresent transmission risk when contact behavior is highly variable. Incorporating IIV alongside mean contact levels may improve infectious disease models and inform public health policies.

**Supplementary Information:**

The online version contains supplementary material available at 10.1186/s12879-026-12940-4.

## Background

Airborne infectious diseases, caused by bacteria or viruses that spread through the air via aerosols and respiratory droplets during contact, are particularly influenced by human contact behavior, that is how often, where, and with whom people interact. These interaction patterns, commonly referred to as social contact patterns, are a central determinant of transmission dynamics.

Since 2006, many studies have demonstrated the importance of social contact patterns for infectious disease modelling [1–3]. A landmark example of contact pattern study is the POLYMOD study, which collected diary-based contact data across eight European countries and provided the first large-scale, cross-national description of age-specific mixing patterns [3]. POLYMOD has since served as a critical baseline for understanding changes in contact behavior during the COVID-19 pandemic [4–7].

From early 2020 onwards, a large body of research documented how contact patterns changed across different phases of the pandemic in countries including China, Germany, Canada, the UK, Netherland, Belgium, Italy, U.S., and Norway [4, 6–16]. These studies reported sharp reductions in contact rates during lockdown periods, followed by partial and heterogeneous recoveries across age groups and settings [10]. Heterogeneity also emerged by immunity-related factors. For example, vaccinated or previously infected individuals in Germany reported more non-household contacts than unvaccinated individuals [17]. And a case-crossover study in England and Wales found increased contacts within 14 days after first vaccination compared with the pre-vaccination period [18].

Epidemic models often rely on method such as contact surveys to parameterize transmission processes, predict epidemic dynamics, and inform intervention strategies [16, 19–24]. However, many epidemic models implicitly assume that contact rates are stable over time or varies mainly between individuals (e.g., by age or occupation) [21, 22, 25–27]. While such approaches capture between-individual heterogeneity they largely overlook within-individual variability in contact behavior over time [28, 29].

In reality, contact behavior reflects both relatively stable characteristics (e.g., age, sex, occupation, or household structure) and time-varying influences (e.g., weekly routines, seasonality, perceived risk, vaccination uptake, and policy measures) [30]. Consequently, individuals’ contact rates can fluctuate substantially from one measurement to the next. Although some variation is attributable to systematic factors (e.g., lockdowns or seasonality), a remaining component is less predictable and can be conceptualized as IIV [31–34].

IIV has been widely studied in psychology and behavioral sciences as an indicator of behavioral flexibility or instability [30, 34–36]. Quantifying IIV in contact behavior is particularly relevant for the COVID-19 pandemic in Germany, which was characterized by rapid alternation between restrictive and relaxed policy phases, multiple epidemic waves, and a staggered vaccination rollout. During such periods, individuals may change not only their average number of contacts but also the consistency of their contact rate over time. Ignoring within-person variability may therefore lead to misleading inferences in epidemic modelling. For instance, two individuals may both have average five daily contacts over a week, but one reports exactly five each day while the other alternates between very few and many. Despite identical mean contact rates, their contact variation values would differ substantially, and epidemic models informed by these patterns would yield different results.

To address this gap, we analyzed 33-wave longitudinal data from the German “COVID Pandemic: Social Contacts and Modelling” (COVIMOD) study to quantify IIV in non-household contact rates and to assess whether IIV varies across vaccination-related periods and policy stringency phases. We operationalize IIV using a residual-based metric (riSD) that captures idiosyncratic fluctuations after accounting for systematic predictors of contact levels.

Specifically, we addressed three research questions: (1) which sociodemographic characteristics are associated with higher riSD, (2) whether riSD changes within individuals around first vaccination using a paired pre/post design, and (3) whether riSD differs between periods of high versus low policy stringency, also using within-person comparisons.

Our analysis proceeds in four steps. First, we fit a generalized linear mixed model (GLMM) for non-household contact counts, adjusting for key socio-demographic, pandemic-related, and time-related covariates to define each individual’s expected contact level. Second, we compute Pearson residuals as deviations of observed contacts from this predicted mean. Third, among participants with at least two observations, we summarize these deviations by the within-person riSD, which serves as a proxy for IIV and captures how strongly an individual’s contact behavior oscillates across survey waves relative to their baseline expectation. Finally, we used riSD as the outcome in subsequent regression models to address our three research questions. We conduct sensitivity analyses using alternative time windows and reporting-interval restrictions.

## Methods

### The COVIMOD contact survey

The COVIMOD contact survey is a 33-wave observational longitudinal study that collected participant contact information between April 2020 and December 2021 during the COVID-19 pandemic. Participants were asked to retrospectively report their contacts of the previous day. In addition, participants were asked to provide quarantine or self-isolation information in the past seven days, COVID-19 test results in the part 14 days, vaccination status, and their perceived seriousness of COVID-19. Age, sex, and other sociodemographic information were also collected. Recruitment was conducted by the market research firm Ipsos, which selected participants from the i-say.com online panel according to age, sex, and regional quotas, ensuring the study sample was representative of the German population in terms of sociodemographic characteristics. All individuals aged 18 years or older with access to the internet and a valid email address were eligible to participate. Adult participants with underage children in their households were invited to report information as proxies for their children, enabling data collection on contacts among children under 18 years of age.

The questionnaire can be found in Supplementary Materials File 1. More details about COVIMOD have also been described elsewhere [4].

COVIMOD used the POLYMOD contact definition: “people met in person with whom you exchanged at least a few words or had physical contact.” Participants reported all contacts from 5:00 a.m. on the previous day to 5:00 a.m. on the survey day, including both household and non-household contacts. Since household contacts were stable, this study focused only on non-household contacts [9]. To minimize potential bias arising from outlier data, we restricted each participant’s non-household contacts to a maximum of 100, consistent with previous studies [4, 9, 17, 37].

### Main covariates

**Sociodemographic characteristics** included age group (0–17, 18–44, 45–64, ≥ 65 years), sex (female, male), household size (1, 2, ≥ 3 persons), and occupation status (employed, retired, homemaker, student, unemployed).

**Pre-existing health issues** were defined as participants or household members either (i) being advised to receive the annual influenza vaccine (waves 1–13), or (ii) belonging to a medium- or high-risk health group (waves 14–33).

**Quarantine or isolation** indicated whether the participant or a household member had been required to quarantine or isolate within the past seven days.

**Self-risk perception** was based on responses to the question, “Coronavirus would be a serious illness for me.” Participants who agreed were classified as high, those who disagreed as low, and those who neither agreed nor disagreed as neutral.

**Infection status** was derived from participants’ questionnaire responses on COVID-19 test results in the preceding 14 days. It was classified into four categories: negative, positive, not tested, and waiting for results. In waves 1–13, test-result information was frequently missing possibly due to limited test availability; these missing values were therefore coded as “not tested.” The main baseline analysis used this recoded infection-status variable. As a sensitivity analysis, we repeated the baseline model using the original (non-recoded) infection-status variable, retaining missing values as missing.

**Survey fatigue** was captured by the number of prior waves participants joined.

### Vaccination variables

**Vaccination status** was derived from participants’ questionnaire responses on whether and when they received COVID-19 vaccination and the number of doses received. Using the self-reported vaccination dates and dose counts, we classified each observation into five categories: vaccine not available (survey waves before 26 December 2020), 0 dose, 1 dose, 2 doses, and ≥ 3 doses. In addition, we calculated **days since first vaccination** as the difference between the survey reporting date and the participant’s reported date of the first dose. When participants did not get vaccination till end of the study, the value of days since first vaccination was set as NA. Last, we computed a **district-level first-dose COVID-19 vaccination coverage**, defined as the cumulative number of individuals who had received at least one vaccine dose divided by the district population on a given date. This variable was evaluated during model development but was not included in the final baseline model because it did not meaningfully improve model fit or alter the main inferences.

### Policy and epidemic context indicators

**COVID-19 7-day incidence trend.** This indicator was provided by the GeoHealth Centre at the Institute for Hygiene and Public Health, University Hospital Bonn. It is calculated from district-level 7-day COVID-19 incidence and reflects the direction and magnitude of recent changes in incidence [38]. The measure ranged from − 89.8 to 89.6, where higher values indicate a steeper increase in incidence, and lower (more negative) values indicate a steeper decrease.

**District-level COVID-19 mortality rate.** District-level COVID-19 mortality was provided by the Robert Koch Institute (RKI), defined as the percentage of reported COVID-19 deaths among confirmed cases by reporting date [39].

**District-level stringency.** We obtained it from the healthcare data platform infas360 [40]. The infas360 German stringency index is methodologically based on the Oxford Stringency Index and captures temporal variation in government response measures [41]. In our data, the index ranged from 0.76 to 67.71, with higher values indicating stricter policies.

**District random effect.** The participant’s district of residence was included as a random effect to account for unobserved district-level heterogeneity, and 384 districts was included.

### Time structure and temporal adjustment

To capture predictable temporal structure in contact behavior, we included:

**Weekday**, categorical indicator for day of week the date that contact happened.

**Day of year**, as a smooth term to allow non-linear seasonality across the calendar year.

**Reporting-date random intercept**, to absorb day-specific common shocks (286 reporting-day levels, constructed as an ordered factor from a numeric day index since the first study date).

**Reporting-week random intercept**, we constructed a reporting-week variable by grouping each survey reporting date into calendar weeks running from Monday to Sunday. Specifically, we assigned each observation the start date (Monday) of its corresponding week. This yielded 65 weekly groups across the study period.

**District-level first-dose vaccination coverage**. We computed a district-level indicator of first-dose COVID-19 vaccination coverage, defined as the cumulative number of individuals who had received at least one vaccine dose divided by the district population on a given date. This variable was evaluated during model development but was not included in the final baseline model because it did not meaningfully improve model fit or alter the main inferences.

Detailed definitions and sources of all variables are provided in the Supplementary Materials File 2.

### Statistical analysis

#### Data structure

Unlike studies specifically designed to investigate IIV with day-to-day data collection over short periods, COVIMOD was designed to capture changes in contact patterns over the course of the pandemic rather than to measure day-to-day variability over short, fixed intervals. The study comprised 33 waves across two years. The average wave duration was 8 days (range: 4–14), and the average inter-wave interval was 11 days (range: 1–29). Because participants could drop out and re-enter, reporting intervals were unequal: among participants with more than one wave, the median inter-observation interval was 16 days (SD = 32), with a maximum of 545 days. Of 7,845 participants, 1,594 (20.3%) contributed data in only one wave, whereas 2,021 (25.8%) contributed data in more than 10 waves.

The outcome of baseline model, the number of non-household contacts, is count data with a highly right-skewed distribution (median = 0, IQR = 0–2, range = 0–100, SD = 63.4). Given the long and unequal reporting intervals and the strong influence of time-varying pandemic conditions, we first estimated each participant’s expected contact level after adjusting for systematic temporal, policy, epidemic, and sociodemographic factors. We then quantified within-person variability using residual dispersion.

### Person residuals and riSD

**Fig. 1:**
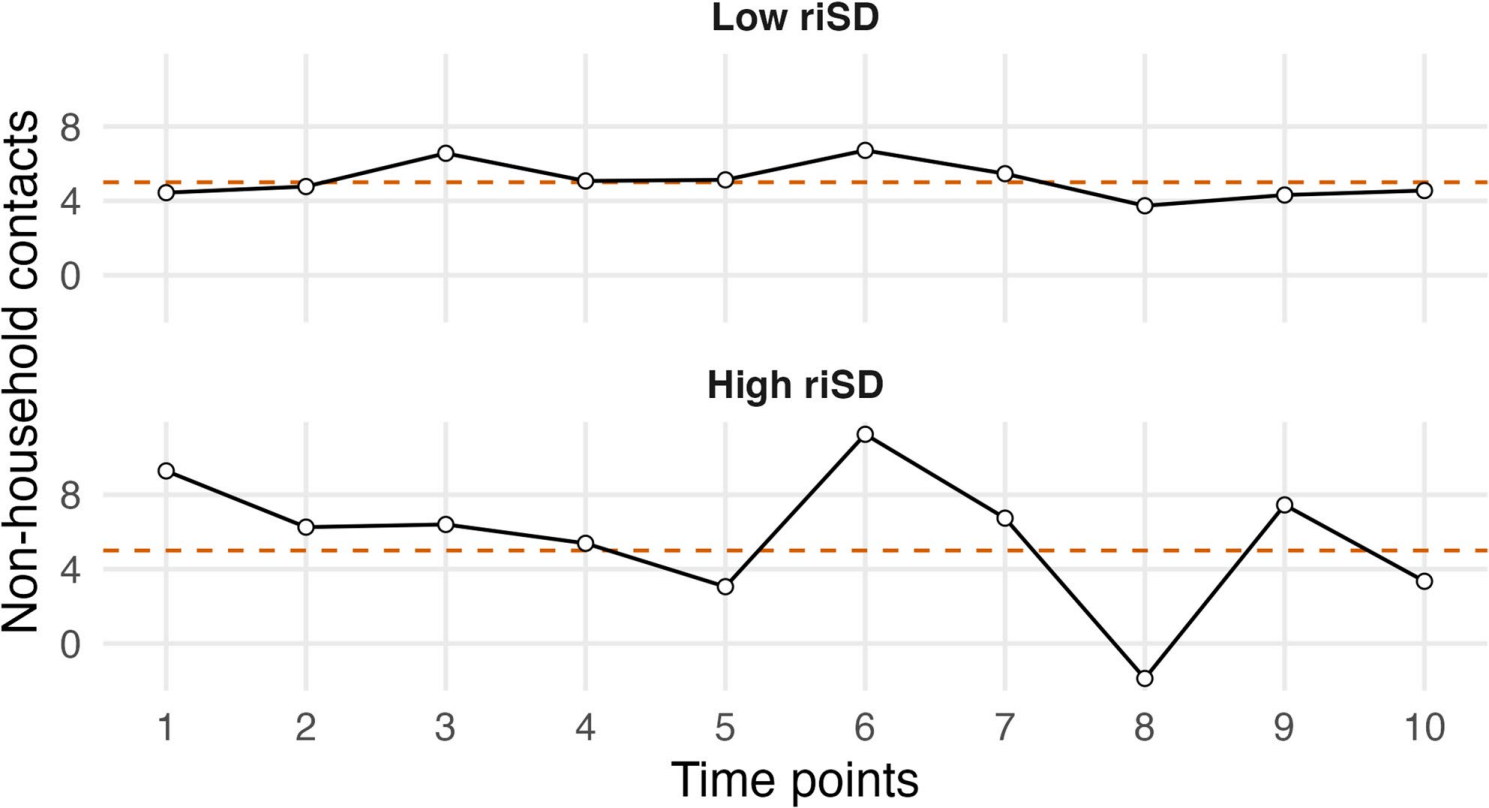
Example illustration of residual individual standard deviation (riSD). Both individuals have the same mean number of contacts, 5 as highlighted by the dashed line, but one is with low riSD (small variability around the mean), while the other shows high riSD (large variability around the mean). Note: this figure is illustrative and does not represent observed trajectories from the study data

To quantify IIV, we applied the two-step residuals method [42]. First, we fitted a baseline generalized linear mixed model to estimate each individual’s expected number of non-household contacts at each survey wave, conditional on observed covariates. We then computed Pearson residuals, which quantify the deviation of the observed contact count from the model-predicted mean at each wave, scaled by the model-implied variability. For each participant with at least two observations, we calculated riSD, defined as the within-person standard deviation of Pearson residuals across waves. Compared with raw within-person variation, this residual-based metric reduces confounding by predictable shifts in contact levels driven by measured covariates and calendar-time effects, as shown in Figure S1. Specifically, riSD is computed as the within-person standard deviation of the Pearson residuals (top row), so the near–zero centering and relatively stable spread of these residuals over calendar time indicate that riSD primarily captures individuals’ idiosyncratic deviations around their expected contact level rather than systematic temporal trends visible in the raw residuals (bottom row). riSD provides a person-level summary of within-person fluctuation in contacts relative to the expected level after adjustment: higher riSD indicates larger wave-to-wave swings (less stable behavior), whereas lower riSD indicates more stable behavior. Figure 1 provides a conceptual illustration to aid interpretation by contrasting two hypothetical individuals with the same average contact level but different variability, highlighting that riSD captures stability/instability rather than mean contact level. Notably, riSD reflects variability in the number of non-household contacts only, and it does not capture changes in who was contacted, the setting, or contact duration.

The formal definition of riSD is:

$$\:{riSD}_{i}=\:\sqrt{\frac{1}{{n}_{i}-1}\:\sum\:_{t=1}^{{n}_{i}}{({r}_{it}-\:\stackrel{-}{{r}_{i}})}^{2}}$$

$$\:{r}_{it}=\:\frac{{y}_{it}-\:{\widehat{\mu\:}}_{it}}{\sqrt{\widehat{Var}\left({y}_{it}\right)}}$$

where $\:{riSD}_{i}$ is the residual individual standard deviation for participant $\:i$; $\:{r}_{it}$is the Pearson residual, and $\:\stackrel{-}{{r}_{i}}\:$is the participant-specific mean residual across their observations; $\:{y}_{it}$ is the observed number of non-household contacts for participant $\:i\:$at observation $\:t$; and $\:\widehat{Var}\left({y}_{it}\right)$ is the variance implied by the fitted model.

### Baseline model selection rationale

We evaluated alternative baseline model specifications during model development, including different sets of pandemic-context indicators (e.g., district-level incidence trend, mortality, and first-dose vaccination coverage), and alternative temporal correlation structures (including an Ornstein–Uhlenbeck correlation term). Candidate models were compared using AIC/BIC and likelihood ratio tests (LRT) where appropriate, while also considering numerical convergence, parameter plausibility, and model diagnostics. We additionally assessed residual patterns and temporal autocorrelation using simulation-based diagnostics. The final model specification was selected as the most parsimonious model that achieved good fit and reduced residual temporal autocorrelation; details of all candidate model structures and model-comparison results are reported in the Supplementary Material File 3.

### Baseline model specification

Let $\:{y}_{it}$ denote the reported number of non-household contacts for participant $\:i$ at observation $\:t$. We fitted a negative binomial generalized linear mixed model with a log link:

$$\:{y}_{it}\:\sim\:{NegBin}_{2}({\mu\:}_{it},\:\theta\:),$$

$$\:\mathrm{log}\left({\mu\:}_{it}\right)=\:{\eta\:}_{it},$$

For the parameterization, the conditional variance is:

$$\:Var\left({y}_{it}\right|\:{\mu\:}_{it})=\:{\mu\:}_{it}+\:\frac{{\mu\:}_{it}^{2}}{\theta\:}$$

The linear predictor $\:{\eta\:}_{it}$ was specified as:

$$\eqalign{ \>\eta {\>_{it}} = \> & \beta {\>_0} + \>\beta {\>^{ \top \>}}{X_{it}} + \>{f_1}\left( {y{d_{it}}} \right) + \>\>{f_2}\left( {S{I_{it}}} \right) + \>\>{f_3}\left( {tren{d_{it}}} \right) + \cr & \>{f_4}\left( {mortali{y_{it}}} \right) + \>\>{b_i} + \>\>{b_{date\left( t \right)}} + \>\>{b_{week\left( t \right)}} + \>{b_{district\left( i \right)}} \cr} $$

Here, $\:{X}_{it}$ includes age group, sex, household size, job status, vaccination status, pre-health conditions, quanrine/isolation status, self-perceived seriousness of COVID-19, recent infection result, joined wave numbers, and weekday. The function $\:{f}_{1}to\:{f}_{4}$ are spline terms for day of year ($\:yd$), district-level stringency index ($\:SI$), district-level COVID-19 incidence trend ($\:trend$), and distict-level COVID-19 mortality ($\:mortaliy$). We included random intercepts for participant ($\:{b}_{i}$), reporting date ($\:{b}_{date\left(t\right)}$), reporting week ($\:{b}_{week\left(t\right)}$), and district ($\:{b}_{district\left(i\right)}$) [43].

After excluding observations with missing values, 7,271 participants with 50,409 observations were included in the baseline model. Participants who joined only one wave were not excluded, as their data still contributed to the model and improved the accuracy of predictions, thereby yielding better residuals for participants who provided data in multiple waves and were included in the next step.

**Fig. 2:**
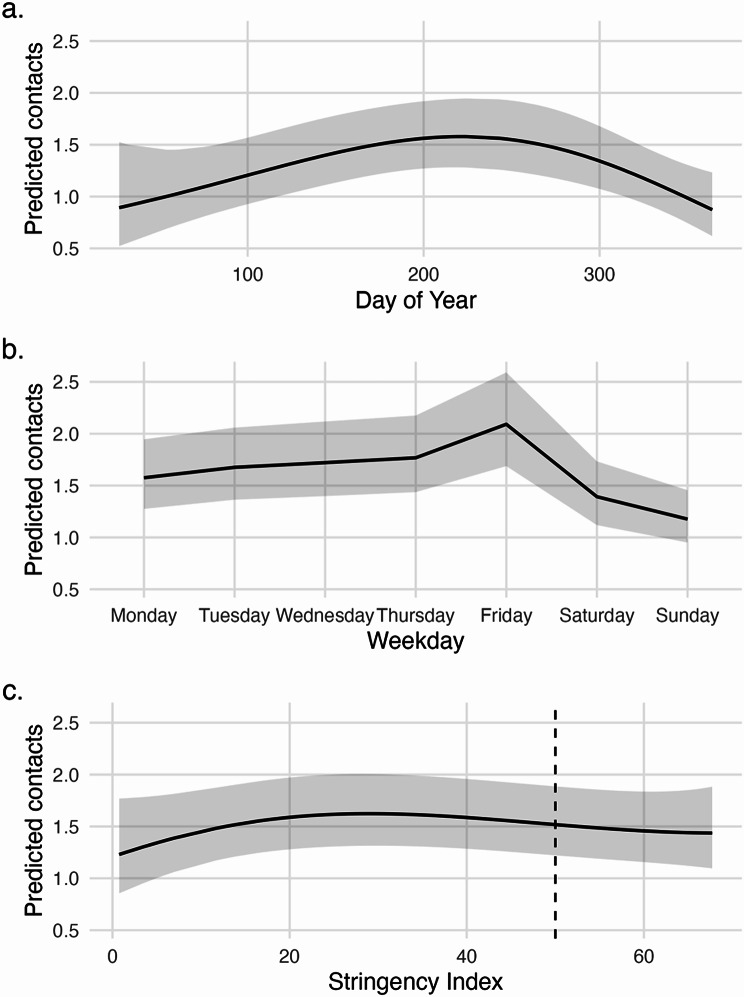
Predicted effects of temporal and policy factors on non-household contacts from the baseline model. Panel (**a**) shows the predicted mean number of non-household contacts across the day of year (seasonality). Panel (**b**) shows predicted contacts by weekday, capturing weekly cycles. Panel (**c**) shows predicted contacts across the district-level stringency index. Predictions are based on the baseline negative binomial mixed model and represent marginal effects while accounting for other covariates in the model. Shaded bands indicate 95% confidence intervals. The vertical dashed line in panel (**c**) (SI = 50) marks the stringency threshold used to define strong versus weak stringency periods in the main analysis

To illustrate the adjustment for time-related and policy effects, Fig. 2 presents predicted non-household contacts from the baseline model by day of year, weekday, and mean district-level stringency index.

### Model specifications of three research questions

First, we examined whether riSD was associated with sociodemographic variables, thereby assessing whether within-individual variability differed systematically across groups.

Second, we assessed whether riSD changed following vaccination. Participants were included if they reported contact behavior within 100 days before and after their first COVID-19 vaccination and contributed at least two observations on both sides of this window. Sensitivity analyses were conducted with narrower time windows (40, 50, and 80 days before and after vaccination, and − 50/+30 days).

**Fig. 3:**
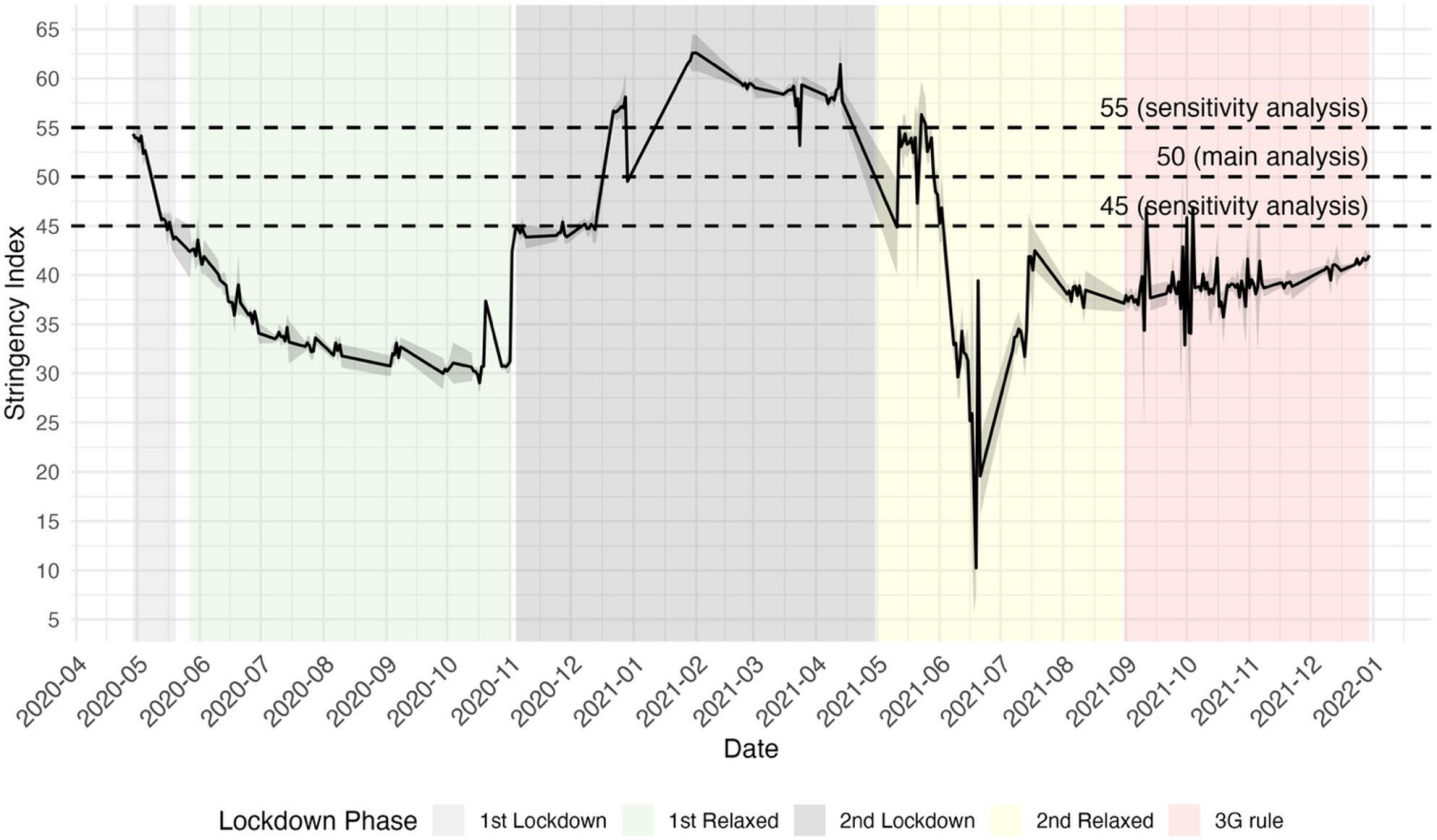
Policy stringency in Germany, April 2020–December 2021. The black line shows the daily mean of the district-level stringency index (0.8–67.7; higher values indicate stricter measures), calculated by averaging the district-level index for each date; the shaded band indicates the 95% CI around the daily mean. Background shading indicates policy phases during the study period: 1st Lockdown (2020-04-29 to 2020-05-20); 1st Relaxed (2020-05-27 to 2020-11-01); 2nd Lockdown (2020-11-04 to 2021-04-30); 2nd Relaxed (2021-05-01 to 2021-08-30); and German “3G rule” (vaccinated, recovered, or tested, implemented in various public spaces, workplaces, and for travel, 2021-08-31 to 2021-12-30). The dashed line at 50 marks the primary threshold used in the main analysis to classify periods as strong (≥ 50) versus weak (< 50) stringency. Dashed green lines at 45 and 55 indicate alternative thresholds used in sensitivity analyses. The district-level stringency index was obtained from infas360

Third, we investigated whether riSD differed between periods of strong (stringency index ≥ 50) and weak (stringency index < 50) social distancing measures. The threshold of 50 was chosen because it coincided with the onset of German lockdown periods (Fig. 3). Sensitivity analyses were conducted using alternative thresholds of 55 and 45.

The model specification for three objectives is as follows.

Let $\:{riSD}_{i}$denote the residual individual standard deviation for participant $\:i$, Because $\:{riSD}_{i}>0$ and is right-skewed, we modelled $\:{riSD}_{i}$ using Gamma distribution with a log link. We additionally weighted each observation by the number of repeated measures used to compute $\:riSD\:\left({n}_{obs,i}\right)$, to reflect that $\:riSD$ is estimated more precisely for participants contributing more waves.

Research question 1: Sociodemographic differences in IIV

$$\:{riSD}_{i}\:\sim\:Gamma\left({\mu\:}_{i},\:\kappa\:\right),\:$$

$$\eqalign{ \>{\rm{log}}\left( {\mu {\>_i}} \right) = \> & \beta {\>_0} + \>\beta {\>_1}AgeGrou{p_i} + \>\beta {\>_2}Se{x_i} + \cr & \>\beta {\>_3}household\>siz{e_i} + \>\beta {\>_4}Jo{b_i}, \cr} $$

with $$\:weights\:{\omega\:}_{i}=\:{n}_{obs,i}.$$

Research question 2: Association between vaccination status and IIV

$$\:{riSD}_{i}\:\sim\:Gamma\left({\mu\:}_{i},\:\kappa\:\right),\:$$

$$\eqalign{ & \>{\rm{log}}\left( {\mu {\>_i}} \right) = \>\beta {\>_0} + \>\beta {\>_1}AgeGrou{p_i} + \>\beta {\>_2}Se{x_i} + \cr & \>\beta {\>_3}household\>siz{e_i} + \>\beta {\>_4}Jo{b_i}\> + \>\beta {\>_5}Vaccin{e_i}, \cr} $$

with $$\:weights\:{\omega\:}_{i}=\:{n}_{obs,i}.$$

Research question 3: Association between stringency and IIV

$$\:{riSD}_{i}\:\sim\:Gamma\left({\mu\:}_{i},\:\kappa\:\right),\:$$

$$\eqalign{ \>{\rm{log}}\left( {\mu {\>_i}} \right) = \> & \beta {\>_0} + \>\beta {\>_1}AgeGrou{p_i} + \>\beta {\>_2}Se{x_i} + \cr \> & \beta {\>_3}household\>siz{e_i} + \>\beta {\>_4}Jo{b_i}\> + \>\beta {\>_5}Stringenc{y_i}, \cr} $$

with $$\:weights\:{\omega\:}_{i}=\:{n}_{obs,i}.$$

### Missing values

The proportion of missing data ranged from 0% (non-household contact number, age group, household size, occupational status, survey date, district, stringency index, COVID-19 incidence trend, mortality, number of waves joined) to 0.4% for sex, 1.1% for vaccination status, 1.8% for quarantine or isolation, 7.3% for self-risk perception, 7.8% for pre-existing health status, and 25.8% for infection status.

Missing values were addressed only for infection status. For wave 1–13, the mean proportion of missing infection-status values per wave was 86.3%, compared with and 0.86% for wave 14–33. The high level of missingness in wave 1–13 were likely caused by limited access to COVID-19 testing during the early pandemic. Therefore, for waves 1–13, missing infection-status values were recoded as “not tested.” After this recoding, the overall missingness in infection status was reduced to 0.6%.

Several sensitivity analyses were performed, (1) restricting the analyses to participants with at least three/four observations when computing riSD; (2) repeating analyses using the original infection-status variable without recoding missing values; (3) restricting the reporting interval to ≤ 100 days and ≤ 50 days when fitting the baseline model.

Stratified analyses (age and sex) and interaction analyses (for the vaccination and stringency analyses) were conducted.

All statistical analyses were conducted using R (version 4.3.3; 2024-02-29) using RStudio [44]. Modeling was performed using the “glmmTMB” [45], and model diagnostics were performed using “DHARMa” package [46].

## Results

### Descriptive statistics

The baseline population comprised 7,845 participants (median age 43 years, IQR 23–62; 50% female). Participants contributed 59,462 observations across 33 waves, with a median of 0 non-household contacts per day (IQR 0–2).

For the analysis of sociodemographic predictors, 5,736 participants with ≥ 2 observations were included. For the vaccination effect on riSD, 1,068 participants who contributed observations within 100 days before and after their first vaccination were included. For the policy-stringency analysis, 2,200 participants contributed data in both periods of strong (SI ≥ 50) and weak (SI < 50) measures were included.

Detailed descriptive statistics for each analysis population are presented in Table 1.

**Table 1:** Description of the study population for the baseline model and for the analytic subsets used in three analyses

Attribute	Baseline model	1st Analysis	2nd Analysis		3rd Analysis	
		social demographic	vaccine effect		stringency effect	
			unvaccinated	vaccinated	Weak stringency index < 50	Strong stringency index ≥ 50
Sample size: responses (participants)	50,409 (7,271	54,693 (5,736)	3,839 (1,068)	3,445 (1,068)	22,164 (2,200)	10,130 (2,200)
Analysis period	April 2020 to December 2021	April 2020 to December 2021	September 2020 to November 2021	January to December 2021	April to December 2020, April to December 2021	April to July2020, October 2020 to May 2021
Non-household contact rate						
Mean (SD)	2.2 (7.9)	2.1 (7.7)	1.6 (6.1)	1.6 (6.5)	2.2 (8.0)	1.6 (6.6)
Median (IQR)	0.0 (0.0–2.0)	0.0 (0.0–2.0)	0.0 (0.0–1.0)	0.0 (0.0–1.0)	0.0 (0.0–2.0)	0.0 (0.0–1.0)
Min | Max	0.0 | 100.0	0.0 | 100.0	0.0 | 100.0	0.0 | 100.0	0.0 | 100.0	0.0 | 100.0
riSD Pearson						
Mean (SD)	NA	0.7 (0.5)	0.5 (0.7)	0.6 (0.9)	0.8 (0.6)	0.6 (0.7)
Median (IQR)	NA	0.7 (0.3-1.0)	0.2 (0.0-0.8)	0.3 (0.0-0.8)	0.7 (0.4–1.1)	0.4 (0.0-0.9)
Min | Max	NA	0.0 | 3.7	0.0 | 5.8	0.0 | 13.5	0.0 | 4.3	0.0 | 5.4
Age						
Mean (SD)	42.6 (22.1)	44.9 (21.8)	50.1 (18.9)		46.9 (21.1)	
Median (IQR)	45.0 (24.0–62.0)	50.0 (27.0–64.0)	55.0 (35.0–66.0)		54.0 (31.0–66.0)	
Min | Max	0.0 | 92.0	0.0 | 92.0	1.0 | 89.0		0.0 | 92.0	
Missing n	0	5	0		0	
Sex						
Male	3,671 (50.5%)	3,047 (48.4%)	584 (54.7%)		1,151 (52 − 3%)	
Female	3,600 (49.5%)	3,195 (51.6%)	484 (45.3%)		1,049 (47,7%)	
Missing n	0	3	0		0	
Household size group						
1	2,129 (29.2%)	1,774 (30.9%)	320 (30.0%)		662 (30.1%)	
2	2,571 (35.4%)	2,078 (36.2%)	413 (38.7%)		760 (34.5%)	
≥ 3	2,580 (35.5%)	1,884 (32.8%)	335 (31.4%)		778 (35.4%)	
Occupational status						
Employed	4,433 (61.0%)	3,733 (59.9%)	646 (60.5%)		1,331 (60.5%)	
Retired	1,560 (21.5%)	1,363 (23.8%)	265 (24.8%)		509 (23.1%)	
Homemaker	341 (4.7%)	266 (4.6%)	45 (4.2%)		109 (5.0%)	
Student	512 (7.0%)	343 (6.0%)	55 (5.1%)		133 (6.0%)	
Unemployed	425 (5.8%)	331 (5.8%)	57 (5.3%)		118 (5.4%)	
Pre-existing health issues, yes	2,374 (32.7%)					
Missing n	0	**—**	—		—	
Quarantine or isolation, yes	1,531 (3.0%)					
Missing n	0	—	—		—	
Self-risk perception						
High	25,605 (50.8%)	**—**	—	—	—	—
Neutral	12,080 (24.0%)	—	—	—	—	—
Low	12,724 (25.2%)	—	—	—	—	—
Missing n	0	—	—	—	—	—
Vaccination status						
not available	18,224 (36.2%)	—	—	—	—	—
0	12,100 (24.0%)	—	—	—	—	—
1	4,758 (9.4%)	—	—	—	—	—
2	8,823 (17.5%)	—	—	—	—	—
>=3	6,504 (12.9%)	—	—	—	—	—
Missing n	0	—	—	—	—	—
Number of Waves joined						
Mean (SD)	7.6 (7.0)	9.6 (7.0)	3.0 (1.0)	3.0 (1.0)	9.6 (6.3)	10.9 (6.1)
Median (IQR)	5.0 (2.0–11.0)	7.0 (4.0–13.0)	3.0 (3.0–4.0)	3.0 (2.0–4.0)	8.0(4.0–12.0)	12.0(6.0–16.0)
Min | Max	1.0 | 30.0	2.0 | 30.0	2.0 | 7.0	2.0 | 7.0	1.0 | 30.0	1.0 | 27.0

Note: Values are n (%) for categorical variables and mean (SD), median (IQR), and min–max for continuous variables. “Responses (participants)” counts survey responses and unique individuals; individuals may appear more than once across waves. Columns for the second analysis compare observations by vaccination status (pre-vaccination = observations prior to the first reported vaccine dose; post-vaccinated = observations after vaccination in participants who were vaccinated; post-unvaccinated = observations from participants who remained unvaccinated), while columns for the third analysis compare observations by district-level stringency index (weak < 50 vs. strong ≥ 50). Abbreviations: “— “: data not applicable; riSD: residual individual-level standard deviation of Pearson residuals; IQR: interquartile range; SD: standard deviation

### Intraclass correlation

The intraclass correlation (ICC) quantifies the proportion of total variance attributable to between-person difference versus within-person differences over time. In the baseline model (including 7,271 participants), the adjusted ICC was 0.655, indicating that 66% of the variance in non-household contacts was attributable to differences between individuals, and 34% to within-person variability over time.

Importantly, 1,535 of these 7,271 participants (21.1%) contributed data from only a single wave, and therefore do not directly inform the within-person variance, which is identified from repeated observations. Consequently, the ICC should be interpreted as primarily reflecting clustering among participants with ≥ 2 observations, and the within-person component as the average within-person variability among those followed over time. Nevertheless, the ICC indicates that within-person variation constitutes a substantial share of overall heterogeneity in non-household contact behavior.

### Sociodemographic predictors of riSD

We modeled the riSD using a Gamma regression with a log link, weighting by each participant’s number of observations. Exponentiated coefficients are reported as mean ratios (MR) of riSD. 5,736 participants were included in this analysis. Compared with adults 18–44 years old, children/adolescents (0–17 years old) had higher within-person variability (MR = 1.13, 95% CI 1.09–1.16). Adults 45–64 years old and ≥ 65 years old were similar to the reference (0.98, 95% CI 0.96–1.00 and 1.00, 95% CI 0.97–1.02, respectively). Sex showed no difference (female vs. male: 1.00, 95% CI 0.99–1.01). Relative to single-person households, 3 + persons had modestly higher riSD (1.05, 95% CI 1.02–1.07), while 2-person households did not differ (1.01, 95% CI 0.99–1.03). Versus the employed, riSD was lower for retired (0.94, 95% CI 0.92–0.96), homemaker (0.89, 95% CI 0.86–0.93), unemployed (0.92, 95% CI 0.88–0.95), and students (0.93, 95% CI 0.90–0.97). Results are shown in Table 2.

**Table 2:** Mean Ratios (MRs) and 95% confidence intervals (CIs) for the association between riSD and covariates from three analyses

	1st Analysis	2nd Analysis	3rd Analysis
Variable	Mean Ratio (95%CI)		
Age group: 18–44	ref	ref	ref
Age group: 0–17	1.13 (1.09–1.16)	1.31 (1.12–1.53)	1.16 (1.11–1.21)
Age group: 45–64	0.98 (0.96-1.00)	1.05 (0.96–1.14)	0.97 (0.94-1.00)
Age group: 65+	1.00 (0.97–1.02)	1.03 (0.93–1.13)	0.97 (0.94-1.00)
Sex: Male	ref	ref	ref
Sex: Female	1.00 (0.99–1.01)	1.06 (0.99–1.13)	1.02 (1.00-1.04)
Household size: 1	ref	ref	ref
Household size: 2	1.01 (0.99–1.03)	0.93 (0.86-1.00)	1.03 (1.00-1.05)
Household size: ≥3	1.05 (1.02–1.07)	0.97 (0.89–1.06)	1.01 (0.98–1.04)
Occupational status: Employed	ref	ref	Ref
Occupational status: Retired	0.94 (0.92–0.96)	1.08 (0.99–1.17)	0.98 (0.95–1.01)
Occupational status: Homemaker	0.89 (0.86–0.93)	1.10 (0.93–1.30)	0.93 (0.88–0.98)
Occupational status: Student	0.93 (0.90–0.97)	0.98 (0.84–1.15)	0.86 (0.82–0.91)
Occupational status: Unemployed	0.92 (0.88–0.95)	1.04 (0.89–1.20)	0.94 (0.89–0.99)
Vaccine: unvaccinated	—	ref	—
Vaccine: vaccinated	—	1.13 (1.06–1.20)	—
Stringency index: ≥ 50	—	—	ref
Stringency index: < 50	—	—	1.36 (1.32–1.39)

Note: The first analysis assessed sociodemographic predictors (age group, sex, household size, occupation). The second analysis assessed vaccination status, adjusted for the same sociodemographic variables. The third analysis assessed policy stringency (stringency index at district level: strong > = 50 vs. weak < 50), also adjusted for sociodemographic. Abbreviations: ref: reference level; “—“: not applicable; riSD = residual-based intra-individual standard deviation

### Vaccination and riSD

Among the 1,068 participants with observations in both windows, riSD was higher in the vaccinated window (days since first dose: 1–100) than in the unvaccinated window (days since first dose: −100 to 0) (MR = 1.13, 95% CI 1.06–1.20).

### Policy stringency and riSD

Among the 2,200 participants with observations in both high- and low-stringency periods, riSD was higher during low stringency (SI < 50) than during high stringency (MR = 1.36, 95% CI 1.32–1.39).

### Sensitivity analyses

In the first sensitivity analysis, we required at least three observations per individual to compute riSD. The resulting estimates were comparable to the main analysis, and conclusions across all three research questions remained unchanged.

In the second sensitivity analysis, we required at least four observations per individual to compute riSD. Results for the first and third research questions were consistent with the main analysis. However, for the second research question, the difference in riSD between the vaccinated and unvaccinated windows was no longer statistically significant (MR = 0.95, 95% CI 0.85–1.07).

For the vaccination-window sensitivity analyses (Sensitivity analysis 3), we retained the ≥ 2-observation criterion but varied the pre/post windows around first dose (± 40, ± 50 days, and ± 80 days; and an asymmetric window of − 50/+30 days). The direction of the estimated vaccination-period contrast was generally positive but less precise. Specifically, the estimate was below 1 in the ± 40-day window (MR = 0.91, 95% CI 0.76–1.11; *n* = 265), whereas the other alternative windows yielded MRs above 1 (± 50 days: MR = 1.09, 95% CI 0.97–1.22; *n* = 599; −50/+30 days: MR = 1.04, 95% CI 0.83–1.31; *n* = 197). The ± 80-day window suggested borderline higher riSD in the vaccinated window (MR = 1.07, 95% CI 1.00–1.16; *n* = 973). Overall, vaccine-window results were sensitive to the chosen time window because of the change of participants numbers, with evidence for higher riSD after vaccination strongest in the main (± 100 days) specification and weakest in the narrow ± 40-day window.

Using the main threshold (SI < 50 vs. SI ≥ 50; *n* = 2,200), riSD was higher during weak stringency compared with strong stringency (MR = 1.36, 95% CI 1.32–1.39). This pattern was robust to alternative thresholds. When defining weak stringency as SI < 55 (*n* = 1780) (Sensitivity analysis 4), the contrast strengthened (MR = 1.47, 95% CI 1.43–1.51). When using a definition of weak stringency (SI < 45 vs. ≥ 45; *n* = 2,370) (Sensitivity analysis 4), the contrast attenuated but remained clearly > 1 (MR = 1.15, 95% CI 1.12–1.17). Overall, riSD was consistently higher in weak-stringency periods across threshold choices, with effect size varying as expected with the cut-off.

To evaluate whether results depended on how infection status was defined, we re-estimated the models using the non-computed infection-status variable (Sensitivity analysis 5). Overall, conclusions were similar across specifications. For the second researc question, the vaccinated-window contrast was essentially unchanged (MR = 1.12, 95% CI 1.05–1.19, *n* = 1,067). For the third research question, riSD remained higher during weak versus strong stringency (MR = 1.23, 95% CI 1.19–1.27, *n* = 2,002).

To assess robustness to the time between repeated observations used to compute riSD, we repeated the analyses after restricting to participants with a reporting interval ≤ 100 days (Sensitivity analysis 6) and ≤ 50 days (Sensitivity analysis 7). Results were broadly consistent with the main analyses.

Full results are provided in Supplementary Materials file 2 Table S2.

### Stratified analyses

For age, we conducted two stratified analyses for participants aged ≥ 65 years and 18–64 years (Table S3). In the ≥ 65-year stratum, occupational categories with very small counts (student, unemployed, homemaker) were combined into an “Other” category. Overall, patterns were broadly consistent with the main analyses. Vaccination remained associated with riSD in both strata (≥ 65 years: MR 1.17, 95% CI 1.05–1.31; 18–64 years: MR 1.03, 95% CI 0.95–1.23). Periods of weaker policy stringency (SI < 50) were associated with higher riSD in both strata (≥ 65 years: MR 1.23, 95% CI 1.17–1.31; 18–64 years: MR 1.23, 95% CI 1.18–1.29).

For sex, analyses were stratified by male and female (Table S3). Results were generally consistent with the main models. The vaccination effect was similar in males and females (male: MR 1.10, 95% CI 1.01–1.20; female: MR 1.11, 95% CI 1.01–1.22). Likewise, weaker policy stringency (SI < 50) was associated with higher riSD in both sexes with male showed stronger effect (males: MR 1.30, 95% CI 1.24–1.37; females: MR 1.15, 95% CI 1.10–1.21).

### Interaction analyses

In interaction models (vaccination * age group; vaccination * sex), we found no evidence that the association between vaccination and riSD differed by age group or sex (all vaccine * age and vaccine * sex interaction terms non-significant; Table S4).

In interaction models (stringency index * age group; stringency index * sex), weaker policy stringency (SI < 50 vs. ≥ 50) remained associated with higher riSD, but the magnitude differed by age group and sex (Table S4). Relative to ages 18–44, the increase in riSD associated with SI < 50 was attenuated in older age groups (SI < 50 * age group 45–64: MR 0.81, 95% CI 0.76–0.86; SI < 50 * age group: ≥65: MR 0.84, 95% CI 0.79–0.90), while the interaction for ages 0–17 was not statistically significant (MR 0.93, 95% CI 0.86–1.02). Compared with males, the association between SI < 50 and riSD was weaker among females (SI < 50×female: MR 0.90, 95% CI 0.86–0.95).

## Discussion

This study shows that within-person variability in non-household contacts (riSD) is structured by sociodemographic factors and shifts with pandemic context. While most prior work has focused on changes in mean contact rates, our results indicate that policy phases and life circumstances also shape the stability of contact behavior over time—an aspect that can matter for transmission when contacts concentrate into intermittent high-contact days.

Children and adolescents (0–17 years) had higher riSD than adults aged 18–44, suggesting more irregular day-to-day contact routines. This may reflect stronger dependence on external schedules (e.g., school attendance, extracurricular activities). Household and occupational roles were also associated with riSD. Larger households (≥ 3 persons) showed slightly higher riSD. In contrast, retired participants, homemakers, students, and unemployed individuals had lower riSD than employed participants, which may reflect more stable routines and fewer externally imposed contact opportunities relative to work-related mobility and scheduling.

Evidence for an association between vaccination and riSD depended on the analytic window. The effect was apparent when using the wider (− 100 to 0 vs. 1 to 100 days) comparison, weaker for ± 80 days, and not evident in narrower windows (± 40 and − 50vs30 days). This pattern is compatible with short-lived or gradually emerging changes in behavior around vaccination rather than an abrupt shift immediately after the first dose. A related pre/post study reported higher odds of any non-household contact within 14 days after the first vaccine dose [47], suggesting that behavioral responses may occur over shorter timescales than our wave-based survey can resolve. Because observations are not densely sampled around the vaccination date, brief fluctuations may be under-captured in our dataset, limiting our ability to detect changes within narrow time windows.

riSD was substantially higher during periods of weaker policy stringency (SI < 50) than during stricter periods (SI ≥ 50). This suggests that relaxation of measures may widen behavioral dispersion, allowing individuals to alternate more between low-contact and high-contact days depending on personal circumstances and opportunities. Interpretation of this association should also consider that the stringency index in Germany tracked broader pandemic phases (e.g., epidemic intensity, variant succession, and vaccination rollout), although we adjusted for multiple time-varying indicators and calendar-time structure. Consistent with studies showing that mean contact rates rise when measures are relaxed and fall when tightened [6, 8, 17, 48], our findings extend this literature by indicating that policy stringency is linked not only to mean contacts but also to within-person instability.

From a modelling perspective, higher within-person variability implies more temporally clustered contacts, which may amplify transmission and sustain spread even when mean contact levels are moderate. Consistent with this, a recent individual-based modelling preprint on workplace contacts shows that explicitly representing day-to-day variability in working contacts can meaningfully change predicted epidemic spread compared with assuming constant contact rates within a week [49]. For public health, our findings suggest that policy relaxation may increase not only contact opportunities but also instability in mixing patterns. Guidance and interventions could therefore benefit from targeting conditions that generate intermittent high-contact days (e.g., episodic workplace mixing or social events), rather than focusing solely on reducing average contact rates. Our study extends the contact-behavior literature by quantifying intra-individual variability (IIV) in non-household contacts, beyond analyses of mean contact levels. We show that policy stringency is associated not only with shifts in average contacts but also with changes in within-person instability (riSD), indicating that behavioral dispersion varies across pandemic phases. Together, these results support modelling frameworks in which transmission risk depends on both mean contact rates and the temporal concentration of contacts within individuals.

Key strengths include a large panel with repeated measures; riSD capturing person-specific fluctuation after extensive adjustment for time-varying context (including epidemic trends and mortality indicators) and random effects; and a paired within-person design for vaccination and stringency analyses that reduces confounding by stable individual traits. Limitations include that riSD summarizes quantitative fluctuation but not partner turnover; varying time spans over which riSD is estimated across individuals; limited temporal resolution around vaccination due to wave-based sampling; recall bias in self-reported contacts; and remaining potential for residual confounding despite time-related controls and autocorrelation diagnostics; although we adjusted for epidemic intensity using incidence-trend indicators and mortality, we did not explicitly account for SARS-CoV-2 variant succession, which may have influenced perceived risk, policy responses, and contact behavior; we assessed changes within $\:\pm\:$100 days of first vaccination but did not model second/third doses occurring within this window, which may partly confound post-first-dose estimates; finally, results may not be directly generalizable to other countries with different cultural, demographic, or policy contexts. In addition, the sample likely under-represents groups without internet access or limited German proficiency and is older than the general German population, so generalizability should be interpreted cautiously.

Future work should link riSD to transmission outcomes and use higher-frequency contact data to clarify behavioral mechanisms underlying instability in mixing. Policies and communication strategies may benefit from considering not only average contact reductions but also stability of behavior.

## Supplementary Information

Below is the link to the electronic supplementary material.

Supplementary Material 1

Supplementary Material 2

Supplementary Material 3

## Data Availability

The datasets used and/or analyzed during the current study are available from the corresponding author on reasonable request.

## Abbreviations

CI: Confidence interval
CoMix: A group of studies on contact behaviour during the COVID-19 pandemic conducted in several European countries
COVID-19: Coronavirus disease caused by SARS-CoV-2
COVIMOD: A German study on contact behaviour during the COVID-19 pandemic
ICC: Intraclass correlation
IIV: Intraindividual variability
Ipsos: A market research company
IQR: Interquartile range
MR: Mean ratios
POLYMOD: A landmark study on contact behaviour
riSD: standard deviation of within individual residuals
SI: Stringency index
