# Supplementary material for: Intraindividual variability in non-household contacts: a German longitudinal study, April 2020–December 2021
Source: BMC Infect Dis. 2026 Feb 21;26:749. doi: 10.1186/s12879-026-12940-4 (PMC13069695; doi:10.1186/s12879-026-12940-4)
Supplement: Supplementary file 2 — Supplementary Material 2 [file 12879_2026_12940_MOESM2_ESM.docx]

**Intraindividual variability in non-household contacts: a German longitudinal study, April 2020–December 2021**

**Additional File 1: COVIMOD Questionnaire**

Chao Xu^1^, Aleksandr Bryzgalov^1^, Johannes Horn^1^, Andrzej K. Jarynowski^3,5^, Vitaly Belik^3^, Veronika K Jaeger^2^, André Karch^2^, Huynh Thi Phuong^2^, Janik Suer^2^, Marlli Zambrano^3^, Steven Schulz^4^, Alejandra Rincón Hidalgo^4^, Ashish Thampi^4^, Richard Pastor^4^, Rafael Mikolajczyk^1^ on behalf of the OptimAgent Consortium

1 Institute for Medical Epidemiology, Biometrics, and Informatics, Martin Luther University Halle-Wittenberg, Halle, Germany

2 Institute of Epidemiology and Social Medicine, University of Münster, Münster, Germany

3 System Modelling Group, Institute of Veterinary Epidemiology and Biostatistics, Freie Universität Berlin, Berlin, Germany

4 Machine Learning Unit, Department of Engineering, NET CHECK GmbH, Berlin, Germany

5 Interdisciplinary Research Institute, Wroclaw, Poland

**Corresponding author:**

Rafael Mikolajczyk,

Institute for Medical Epidemiology, Biometrics, and Informatics, Medical Faculty of the Martin Luther University Halle-Wittenberg,

Magdeburger Straße 20,

06112,

Halle (Saale), Germany

Email: rafael.mikolajczyk@uk-halle.de

Q1. What is your age in years?

YEAR/MONTH.

Q2. Which of the following describes how you think of yourself?

1. Male
2. Female
3. In another way
4. Prefer not to answer

Q3. Not including you, how many other people live in your household? By household, we mean anyone living at the same address as you, that you share a kitchen with.

None

1

2

3

4

5

6

7

8

9

10

11 or more

Q4. What is your occupation? If retired or unemployed, please indicate the category closest to your previous occupation.

In which of the following categories does your occupation fall?

Legislators, senior officials, and managers

Legislators and senior officials corporate managers

Directors and chief executives Production and operations department managers

Production and operations department managers in agriculture, hunting, forestry, and fishing Production and operations department managers in manufacturing

Production and operations department managers in construction

Production and operations department managers in wholesale and retail trade Production and operations department managers in restaurants and hotels

Production and operations department managers in transport, storage, and communications Production and operations department managers in business services

Production and operations department managers in personal care, cleaning, and related services

Production and operations department managers not elsewhere classified

Other department managers

Finance and administration department managers

Personnel and industrial relations department managers

Sales and marketing department managers

Advertising and public relations department managers

Supply and distribution department managers

Computing services department managers

Research and development department managers

Other department managers not elsewhere classified

General managers

General managers in agriculture, hunting, forestry/ and fishing

General managers in manufacturing

General managers in construction

General managers in wholesale and retail trade

General managers of restaurants and hotels

General managers in transport, storage, and communications

General managers of business services

General managers in personal care, cleaning, and related services

General managers not elsewhere classified

Physical, mathematical, and engineering science professionals

Physicists, chemists, and related professionals

Mathematicians, statisticians, and related professionals

Computing professionals

Architects, engineers, and related professionals Architects, town, and traffic planners

Civil engineers

Electrical engineers

Electronics and telecommunications engineers

Mechanical engineers

Chemical engineers

Mining engineers, metallurgists, and related professionals

Cartographers and surveyors

Architects, engineers, and related professionals not elsewhere classified

Life science and health professionals

Life science professionals

Health professionals (except nursing)

Nursing and midwifery professionals

Teaching professionals

Other professionals

Business professionals

Legal professionals

Archivists, librarians and related information professionals

Social science and related professionals

Writers and creative or performing artists

Religious professionals

Physical and engineering science associate professionals

Physical and engineering science technicians

Computer associate professionals

Optical and electronic equipment operators Ship and aircraft controllers and technicians

Safety and quality inspectors

Life science and health associate professionals

Teaching associate professionals

Other associate professionals

Finance and sales associate professionals Business services agents and trade brokers Administrative associate professionals

Customs, tax, and related government associate professionals

Police inspectors and detectives

Social work associate professionals

Artistic, entertainment and sports associate professionals

Religious associate professionals

Clerks

Office clerks

Customer services clerks

Personal and protective services workers

Travel attendants and related workers

Housekeeping and restaurant services workers

Personal care and related workers

Other personal services workers

Protective services workers

Models, salespersons, and demonstrators

Skilled agricultural and fishery workers

Skilled agricultural and fishery workers

Craft and related trades workers

Extraction and building trades workers

Metal, machinery, and related trades workers

Metal moulders, welders, sheet-metal workers, structural - metal preparers, and related trades workers

Blacksmiths, tool-makers and related trades workers

Machinery mechanics and fitters

Electrical and electronic equipment mechanics and fitters

Precision, handicraft, printing, and related trades workers

Precision workers in metal and related materials

Potters, glass-makers and related trades workers

Handicraft workers in wood, textile, leather and related materials

Printing and related trades workers

Other craft and related trades workers

Food processing and related trades workers

Wood treaters, cabinet-makers and related trades workers

Textile, garment, and related trades workers

Pelt, leather, and shoemaking trades workers

Plant and machine operators and assemblers

Plant and machine operators and assemblers

Elementary occupations

Sales and services elementary occupations

Agricultural, fishery and related labourers

Labourers in mining, construction, manufacturing, and transport

Armed forces

Armed forces Did not work before

Unemployed but looking for a job

Unemployed and not looking for a job / Long-term sick or disabled

Pupil /Student/ in full time education

Housewife

Retired

Q5. Have you been vaccinated against the virus that causes Coronavirus (COVID-19)?

1. Yes
2. No
3. Don’t know

Q6. Please provide the dates you received your vaccination(s) against the virus that causes COVID-19.

1. Date:
2. Don’t know

Q7. Do you or any other member of your household belong to a risk group for which the yearly flu vaccination is recommended?

1. Yes
2. No
3. Don’t know

Q8. Are you or any other household member in a medium risk group, meaning you could have serious symptoms if you contracted Coronavirus (COVID-19)?

Medium risk groups include individuals who: have a lung condition that is not severe (such as asthma, COPD, emphysema or bronchitis), have heart disease (e.g. heart failure), have diabetes, have chronic, kidney or liver disease, have a condition affecting the brain or nerves (such as Parkinson's disease, motor neurone disease, multiple sclerosis or cerebral palsy), have a condition that means they have a high risk of getting infections, are taking medicine that can affect the immune system (such as low doses of steroids), have a BMI of 40 or above, and/or are pregnant.

1. Yes
2. No
3. Don’t know
4. Prefer not to answer

Q9. Are you or any other household member in in a high risk group, meaning you/they could have serious symptoms if you contracted Coronavirus (COVID-19)?

High risk groups include individuals who: have had an organ transplant, undergoing cancer treatment, have blood or bone marrow cancer, have had a bone marrow or stem cell transplant in the past 6 months, are taking immunosuppressant medicine or high doses of steroids, have a severe lung condition (such as cystic fibrosis, severe asthma or severe COPD), have a condition that makes risk of getting infections higher (e.g. SCID or sickle cell), and/or are pregnant and have a serious heart condition.

1. Yes
2. No
3. Don’t know
4. Prefer not to answer

Q10. You may have been asked to do so or have voluntarily decided to take various measures against the coronavirus (Covid-19). Please think back to the past seven days and select the appropriate answer for each of the actions listed below. In the last seven days have you or has anyone in your household been in isolation or quarantine due to coronavirus (Covid-19)?

1. Yes
2. No
3. Prefer not to say

Q11. To what extent do you agree or disagree with each of the following statement, Coronavirus would be a serious illness for me

1. Strongly agree
2. Tend to agree
3. Neither agree nor disagree
4. Tend to disagree
5. Strongly disagree
6. Don’t know
