# Supplementary material for: Intraindividual variability in non-household contacts: a German longitudinal study, April 2020–December 2021
Source: BMC Infect Dis. 2026 Feb 21;26:749. doi: 10.1186/s12879-026-12940-4 (PMC13069695; doi:10.1186/s12879-026-12940-4)

**Intraindividual variability in non-household contacts: a German longitudinal study, April 2020–December 2021**

**Additional File 3**: **model selection, fit summary, and diagnostics**

Chao Xu^1^, Aleksandr Bryzgalov^1^, Johannes Horn^1^, Andrzej K. Jarynowski^3,5^, Vitaly Belik^3^, Veronika K Jaeger^2^, André Karch^2^, Huynh Thi Phuong^2^, Janik Suer^2^, Marlli Zambrano^3^, Steven Schulz^4^, Alejandra Rincón Hidalgo^4^, Ashish Thampi^4^, Richard Pastor^4^, Rafael Mikolajczyk^1^ on behalf of the OptimAgent Consortium

1 Institute for Medical Epidemiology, Biometrics, and Informatics, Martin Luther University Halle-Wittenberg, Halle, Germany

2 Institute of Epidemiology and Social Medicine, University of Münster, Münster, Germany

3 System Modelling Group, Institute of Veterinary Epidemiology and Biostatistics, Freie Universität Berlin, Berlin, Germany

4 Machine Learning Unit, Department of Engineering, NET CHECK GmbH, Berlin, Germany

5 Interdisciplinary Research Institute, Wroclaw, Poland

**Corresponding author:**

Rafael Mikolajczyk,

Institute for Medical Epidemiology, Biometrics, and Informatics, Medical Faculty of the Martin Luther University Halle-Wittenberg,

Magdeburger Straße 20,

06112,

Halle (Saale), Germany

Email: rafael.mikolajczyk@uk-halle.de

**Model selction**

Baseline contact counts were modeled using a negative binomial mixed-effects model (NB2), with random intercepts for participant, calendar day, calendar week, and district, and spline terms for seasonal timing (day of year), district-level stringency, and the background epidemic trend.

Our model selection strategy was to first specify the fixed and random effects and then select the best-fitting model. Starting from this model, we added contextual district-level variables (e.g., COVID-19 incidence trends, mortality, and first-dose vaccination coverage) to assess whether they significantly improved model fit.

First, we fitted four candidate models as follows:

Model 1: weekday was included as a smooth, nonlinear effect

$$m_{baseli{ne}_{1}}\sim age group+sex+household site+occupation+vaccination status+pre-health status+quarantine+self reisk+infection status+wave_{n}+ns\left( weekday \right)+bs\left( yd \right)+bs\left( stringency index \right)+\left( 1 | participant id \right)+\left( 1 | dt_{fac} \right)+\left( 1 | week \right)+ (1|district)$$

Model 2: weekday was included as a categorical (factor) variable

$$m_{baseli{ne}_{2}}\sim age group+sex+household site+occupation+vaccination status+pre-health status+quarantine+self reisk+infection status+wave_{n}+weekday+bs\left( yd \right)+bs\left( stringency index \right)+\left( 1 | participant id \right)+\left( 1 | dt_{fac} \right)+\left( 1 | week \right)+ (1|district)$$

Model 3: weekday was included as circular variable

$$m_{baseline3}\sim age group+sex+household site+occupation+vaccination status+pre-health status+quarantine+self reisk+infection status+wave_{n}+cubic\left( weekday \right)+bs\left( yd \right)+bs\left( stringency index \right)+\left( 1 | participant id \right)+\left( 1 | dt_{fac} \right)+\left( 1 | week \right)+ (1|district)$$

Model 4: model 2 without including wave_n

$$m_{baseli{ne}_{4}}\sim age group+sex+household site+occupation+vaccination status+pre-health status+quarantine+self reisk+infection status+weekday+bs\left( yd \right)+bs\left( stringency index \right)+\left( 1 | participant id \right)+\left( 1 | dt_{fac} \right)+\left( 1 | week \right)+ (1|district)$$

Comparison of the four candidate models based on AIC and BIC

|  | Model 1 | Model 2 | Model 3 | Model 4 |
| --- | --- | --- | --- | --- |
| AIC | 149321.2 | 149298.6 | 149348.8 | 149472.5 |
| BIC | 149647.9 | 149651.7 | 149666.6 | 149816.8 |

We then selected Model 2 as the best-performing structural model. Building on this specification, we fitted three baseline models by additionally including district-level contextual variables: (i) COVID-19 incidence trend, (ii) mortality, and (iii) first-dose vaccination coverage.

Model 2 + district-level incidence trend

$$m_{baseli{ne}_{2+trend}}\sim age group+sex+household site+occupation+vaccination status+pre-health status+quarantine+self reisk+infection status+wave_{n}+weekday+bs\left( yd \right)+bs\left( stringency index \right)+\left( 1 | participant id \right)+\left( 1 | dt_{fac} \right)+\left( 1 | week \right)+ \left( 1 | district \right)+trend$$

Model 2 + district-level mortality

$$m_{baseli{ne}_{2+mortality}}\sim age group+sex+household site+occupation+vaccination status+pre-health status+quarantine+self reisk+infection status+wave_{n}+weekday+bs\left( yd \right)+bs\left( stringency index \right)+\left( 1 | participant id \right)+\left( 1 | dt_{fac} \right)+\left( 1 | week \right)+ \left( 1 | district \right)+mortality$$

Model 3 + district-level foirst dose vaccine coverage

$$m_{baseli{ne}_{2+vaccine}}\sim age group+sex+household site+occupation+vaccination status+pre-health status+quarantine+self reisk+infection status+wave_{n}+weekday+bs\left( yd \right)+bs\left( stringency index \right)+\left( 1 | participant id \right)+\left( 1 | dt_{fac} \right)+\left( 1 | week \right)+ \left( 1 | district \right)+vaccine$$

We then compared Model 2 with each of the three extended models separately using likelihood ratio tests (LRTs).

|  | Pr(>Chisq) |
| --- | --- |
| Model 2 |  |
| Model 2 + trend | 0.04984 * |

|  | Pr(>Chisq) |
| --- | --- |
| Model 2 |  |
| Model 2 + mortality | 0.04085 * |

|  | Pr(>Chisq) |
| --- | --- |
| Model 2 |  |
| Model 2 + vaccine | 0.4977 |

Because adding the incidence trend and mortality terms significantly improved model fit (p = 0.0498 and p = 0.0409, respectively), we fitted an additional model that included both terms simultaneously.

$$m_{baseli{ne}_{2+trend+mortality}}\sim age group+sex+household site+occupation+vaccination status+pre-health status+quarantine+self reisk+infection status+wave_{n}+weekday+bs\left( yd \right)+bs\left( stringency index \right)+\left( 1 | participant id \right)+\left( 1 | dt_{fac} \right)+\left( 1 | week \right)+ \left( 1 | district \right)+trend+mortality$$

|  | Pr(>Chisq) |
| --- | --- |
| Model 2 + trend |  |
| Model 2 + trend + mortality | 0.04032 * |

|  | Pr(>Chisq) |
| --- | --- |
| Model 2 + mortality |  |
| Model 2 + trend + mortality | 0.0492 * |

Because including both terms significantly improved model fit, we selected the model containing both incidence trend and mortality as the final baseline model.

**Model diagnositcs**

**Check for converge**


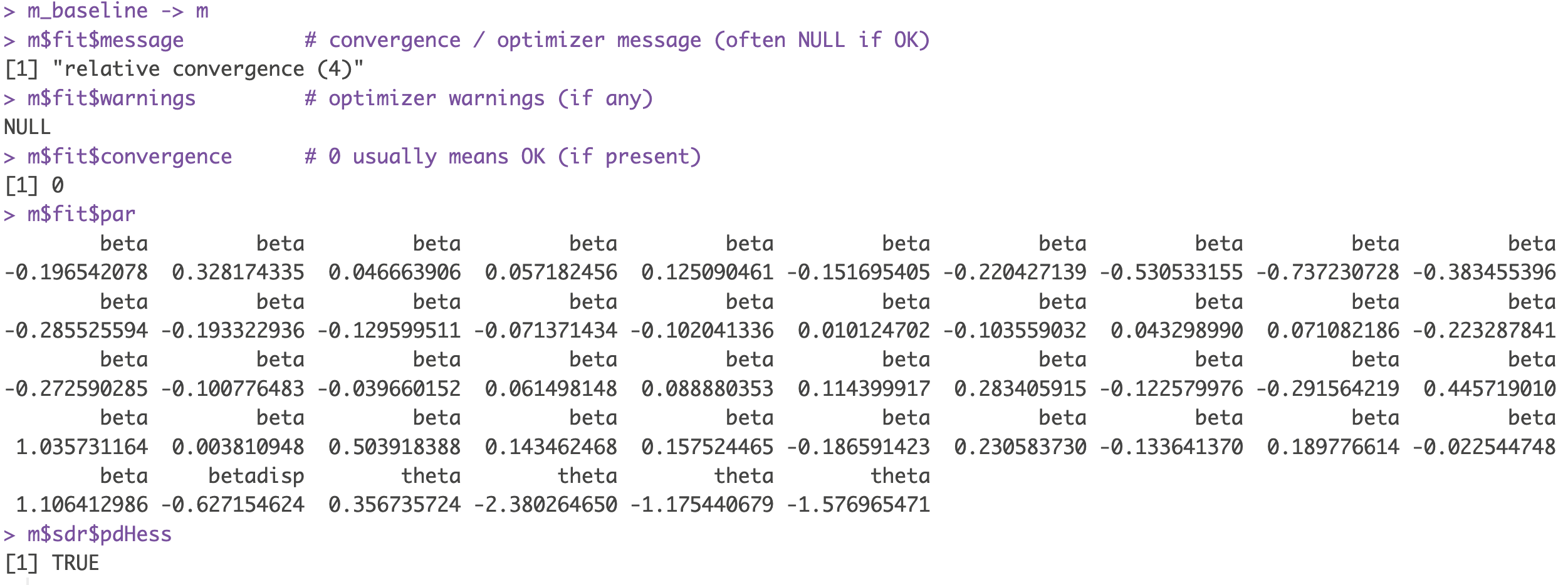


The baseline model converged successfully (convergence code = 0), with no optimizer warnings and a positive-definite Hessian.

**Check for Multicollinearity**


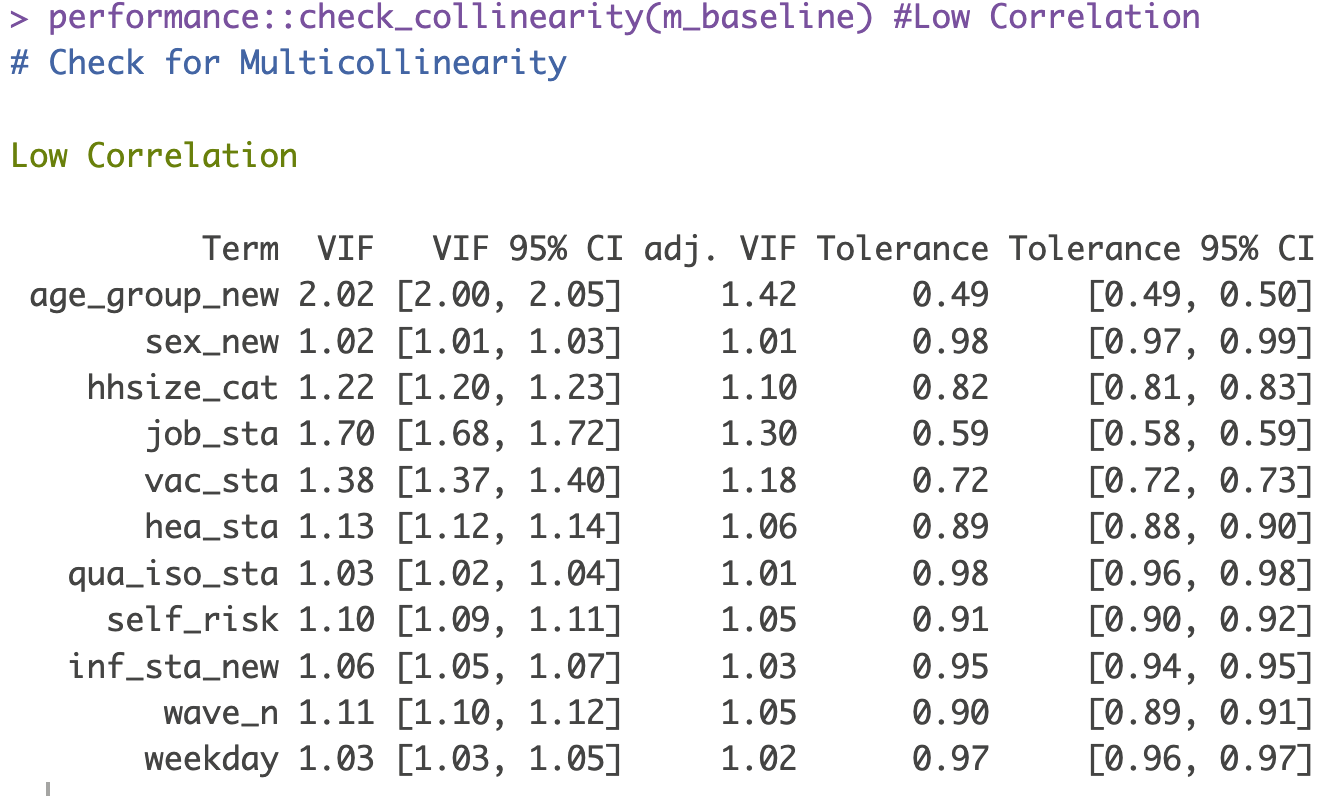


**Check residuals**


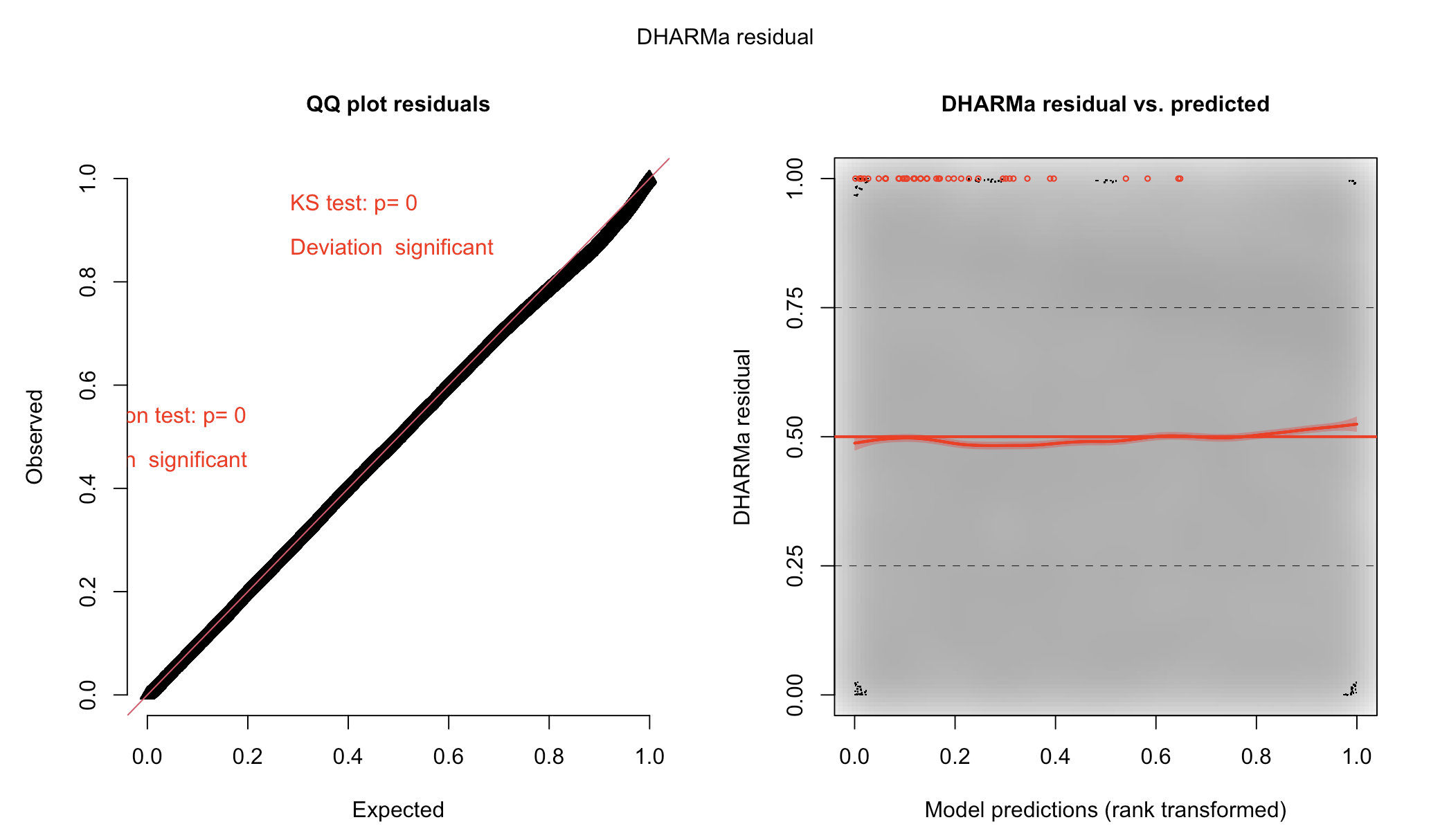


DHARMa diagnostics indicated a small but statistically significant deviation from uniformity (KS test p < 0.001), which is expected in large samples. Visual inspection of the residual QQ plot and residuals versus predicted values showed no pronounced systematic pattern, suggesting that model fit was generally adequate.


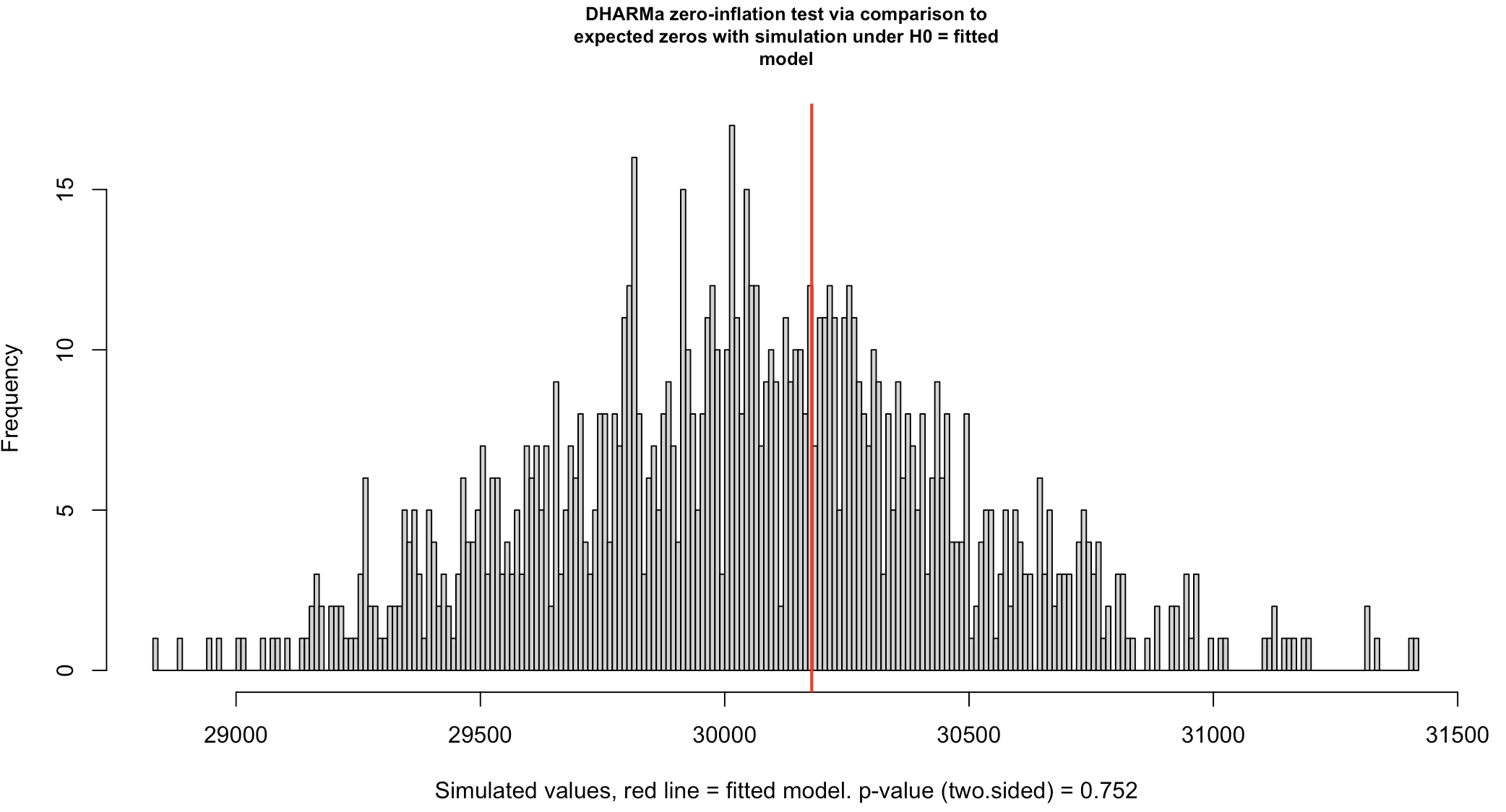


DHARMa diagnostics suggested no evidence of zero inflation beyond that expected under the fitted negative binomial model (zero-inflation test, p = 0.752).


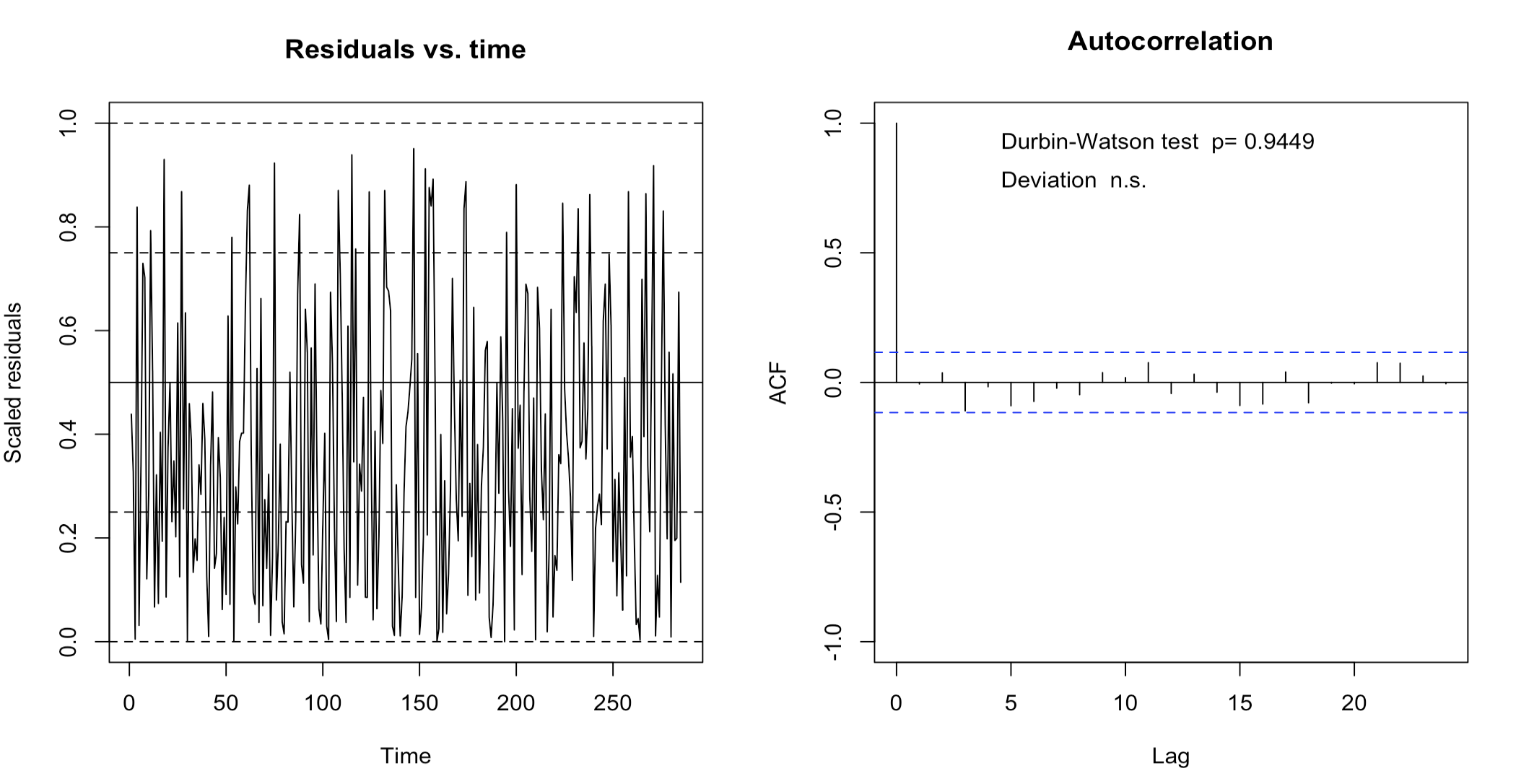


After accounting for calendar-time structure (daily and weekly random effects), scaled DHARMa residuals showed no evidence of temporal autocorrelation (Durbin–Watson DW = 2.01, p = 0.945; ACF within confidence bounds).

In conclusion, we fitted a negative binomial mixed-effects model (NB2; log link). The model was fitted to **N = 50,409** observations from **7,271** individuals across **65** weeks and **384** districts. Model fit statistics were **AIC = 149,294.5, BIC = 149,700.6, logLik = −74,601.24,** and the NB2 dispersion parameter (**θ**) was **0.534**. Convergence diagnostics indicated a stable fit (convergence code = 0; positive-definite Hessian; no optimizer warnings). DHARMa checks suggested no evidence of zero inflation (p = 0.752) and no residual temporal autocorrelation (Durbin–Watson **DW = 2.01**, p = 0.945). Although the DHARMa uniformity test indicated statistically detectable deviation (KS p < 0.001), residual-versus-predicted plots showed no pronounced systematic patterns, consistent with adequate overall fit.


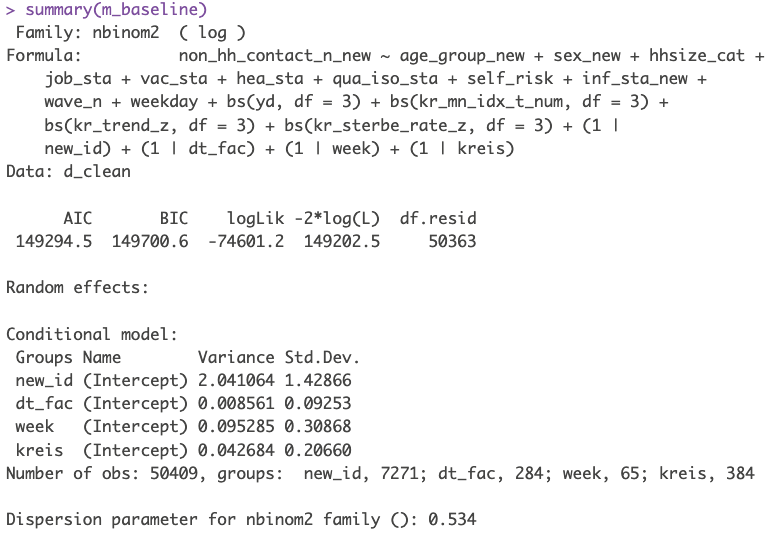

Supplement: Supplementary file 3 — Supplementary Material 3 [file 12879_2026_12940_MOESM3_ESM.docx]
